# An Adaptive Algorithm and Additively Manufactured Punch Used to Form Aluminum Sheet Metal Parts

**DOI:** 10.3390/ma16103704

**Published:** 2023-05-12

**Authors:** Vlad Andrei Ciubotariu, Cosmin Constantin Grigoras, Valentin Zichil, Ana-Maria Rosu

**Affiliations:** 1 Department of Industrial Systems Engineering and Management, “Vasile Alecsandri” University of Bacău, 157 Calea Mărăşeşti, 600115 Bacău, Romania; vlad.ciubotariu@ub.ro; 2 Department of Engineering and Management, Mechatronics, “Vasile Alecsandri” University of Bacău, 157 Calea Mărăşeşti, 600115 Bacău, Romania; 3 Department of Chemical and Food Engineering, “Vasile Alecsandri” University of Bacău, 157 Calea Mărăşeşti, 600115 Bacău, Romania; ana.rosu@ub.ro

**Keywords:** adaptive algorithm, Python-to-C++ bridge, computer vision, 3D-printed punch, topological study

## Abstract

Self-adaptive mechanisms are gaining momentum in industrial processes. It is understandable that as the complexity increases, the human work must be augmented. Considering this, the authors have developed one such solution for the punch-forming process, using additive manufacturing, i.e., a 3D-printed punch, to draw into shape 6061-T6 aluminum sheets. This paper aims to highlight the topological study used to optimize the punch form shape, the methodology of the 3D printing process, and the material used. For the adaptive algorithm, a complex Python-to-C++ bridge was created. It was necessary as the script has computer vision (used for calculating stroke and speed), punch force, and hydraulic pressure measurement capabilities. The algorithm uses the input data to control its subsequent actions. Two approaches are used in this experimental paper, a pre-programmed direction and an adaptive one, for comparison purposes. The results, namely the drawing radius and flange angle, were statistically analyzed using the ANOVA methodology for significance. The results indicate significant improvements when using the adaptive algorithm.

## 1. Introduction

When exceeding the yielding limit of the metallic material, residual strains occur. Changes in the part’s shape appear after the forming forces act. Different specific factors and parameters also influence the metal parts, resulting from various forming processes [1,2]. The deformation of any element can be described based on the β ratio between the strains or the α ratio between the stresses. These ratios will be constant in the case of proportional deformations [3].

The main influencing factors in the plastic forming processes of sheet metals are the following: chemical composition and material structure; mechanical properties of the material; equipment—tools, machines, devices; semi-finished part—geometry, surface quality; the complexity of the finished part’s geometry—shape, dimensions; forming conditions—forming operation, temperature, displacement rate, lubrication conditions [1,4,5].

The cold bending of sheet metal is an elastoplastic process with residual strains appearing in the material during forming [4]. Depending on the part geometry and processing techniques, bending can be performed in either a V or U shape, or freely between the rollers.

The following relations give the principal strain in the case of bending:

(1) $$\varepsilon_{1}=-\varepsilon_{3}=ln\left(1+\frac{1}{(2R/t)+1}\right)\neq 0,\;\;\varepsilon =0$$

The analysis of Equation (1) highlights that the sheet metals are thinning during forming as the radius R decreases. As the radius R decreases, the principal strains ε_1_ and ε_3_ increase, the sheet metal’s thinning t is more and more pronounced, and, in the end, the failure occurs. This means of drawing the blank during bending creates a state of uneven stresses on the thickness of the material, to which an uneven strain state corresponds.

The accuracy of the sheet metal parts obtained by bending and drawing depends mainly on the following factors: the geometry of the parts, the quality of the materials and semi-finished parts, the quality of the forming tools, and the forming conditions.

**Figure 1:**
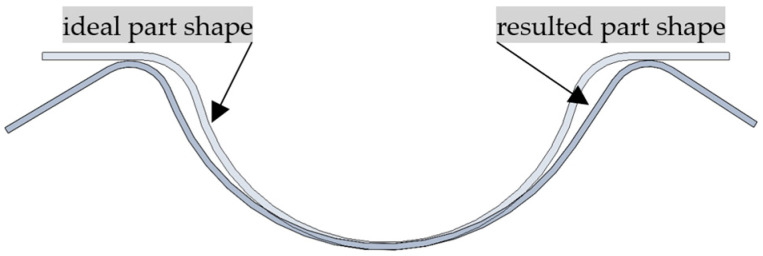
U-shaped metal part obtained by cold punch forming, indicating the ideal comparative to the resulting part shape, to highlight the effect of the springback effect.

The most important phenomenon in the instability of the shape and dimensions of the sheet metal parts, obtained by cold plastic forming, is the springback phenomenon specific to both bending and drawing processes [1,2]. The springback in the cold plastic forming of the sheet metals is given by the difference between the final shape of the part and the shape obtained at the end of the punch stroke. After the withdrawal of the tools, the formed part undergoes significant changes in shape, which means that springback is an additional material deformation that occurs during rebound [6,7,8], as indicated in Figure 1. The analysis of stress/strain diagrams leads to the conclusion that the intensity of springback will increase with the material strength [9,10,11,12,13].

The rebound process is elastic, but a secondary plastic flow can also occur due to the bending and straightening around the edges of the die and punch. When metal sheets are bent, springback is manifested by the angle change and the formed part’s curvature [1,2,6,7,8]. The parameters that characterize the effect of springback are the springback angle θ and the radius of curvature R after the rebound. These parameters depend on the following factors:

- the physical and mechanical properties of the material;
- the shape of the part and the thickness of the sheet metal;
- the bending radius, working scheme, and bending process used.

Unlike bending, where springback is expressed by an angle, or the modification of the part’s curvature when drawing, the parameters of springback are the difference in the height or the change in the radius of curvature. Generally, the springback is positive, but negative values can be recorded in the case of a high clamping force, and the bending radius of the part is less than that of the punch.

Because the phenomenon of springback is favored by the material’s elastic properties, any factor that decreases the ratio between elastic and plastic strain determines the reduction in the springback intensity. Therefore, materials with a lower yield strength and Young’s modulus, higher hardening, a normal anisotropy index, a higher clamping force value, a different punch geometry, or a lubricated environment between the die and the sheet metals can reduce the intensity of the phenomenon [1,2,6,7,8,9,14,15].

Therefore, the tools specific to the plastic forming process can be manufactured in a specific way to satisfy the requirements to decrease the intensity of the springback effect [16,17,18]. A different approach is that of producing some tools using additive manufacturing [19,20].

The term “additive manufacturing” or “rapid prototyping” describes the manufacturing of parts by successively adding layer-to-layer material. This method allows for the production of components with ready-assembled static and mobile elements in a single manufacturing session [20,21,22].

The part is modeled in a digital format. Thus, it can be easily stored, copied, or transferred to the system that ensures its construction. For this purpose, the virtual model is sliced into horizontal parallel sections. Each of these sections is then materialized layer upon layer with the help of specialized equipment that allows the transfer from the digital format to the material form.

The materials used in additive manufacturing include numerous polymers, metals (aluminum, steel, titanium, bronze, silver, gold), quartz, ceramics, and many composite materials [20,21,22,23,24].

Unlike classical technologies, additive manufacturing has several significant advantages, such as the following:

- it allows the fabrication of parts with complex structures that cannot be achieved by other methods/means and offers the possibility of improving the performance and functionality of the products, adapting the products to the individual needs of consumers;
- it allows the customization of any object made, covering, at the level of the production method, both the production of series and personalized objects, thus addressing the two major markets;
- it allows savings of material, energy, and human resources.

If, by classical technologies, in many cases, the part is made by removing material, additive manufacturing involves the agglomeration of matter particles, thus using less material. Thus, material not used during a manufacturing session can be reused in a proportion of over 90%. 

It is estimated that the fabrication of layer-by-layer parts can reduce material costs and requirements by up to 90% [25]. The elimination of some stages in the production chain (since the parts can be made as soon as they have been designed, without the need to build parts or prototypes), the use of smaller quantities of material, and the creation of lighter objects also imply a reduction in the amount of energy consumed during the manufacturing procedure. 

Additive manufacturing technologies allow for rapid adaptation to user needs and the creation of new production options outside the factory, such as mobile production units located near the material source. 

The devices most used in additive manufacturing are grouped under the generic name “3D printers”, and the most widespread groups of technologies used refer to photopolymerization, selective sintering, or extrusion.

One of the most widespread means of performing additive manufacturing is 3D filament printing. This process requires a material to be extruded through a nozzle on a specific Cartesian interpolated path, thus forming successive layers. The extrusion of heated thermoplastic materials represents some variations of this process with a limited dosage [20,21,22].

The process is an economic one compared to the other additive processes. The technology is clean and easy to carry out. Moreover, the parts have good structural properties.

This technology allows filaments made of thermoplastic materials for FFF or the supply of flakes—through push screws—and liquids or slurries through plungers for FDM. Systems intended for material extrusion may also be fitted with one or more additional heads. Thus, they can be fed simultaneously or in parallel with the primary and support materials.

## 2. Methodology

### 2.1. General Methodology

Punch-forming is used at an industrial scale for punching, slitting, forming, tapping, or marking, with the punching tool usually manufactured from steel. 

The adaptive punch-forming process proposed by the authors in this experimental study seeks to highlight the use of an optimized 3D-printed punch along with a comparison between a standard pre-programmed and an adaptive one. It implies deforming aluminum blanks while measuring the punch force, speed, stroke, clamping system pressure, drawing radius, flange angle, and process time.

**Figure 2:**
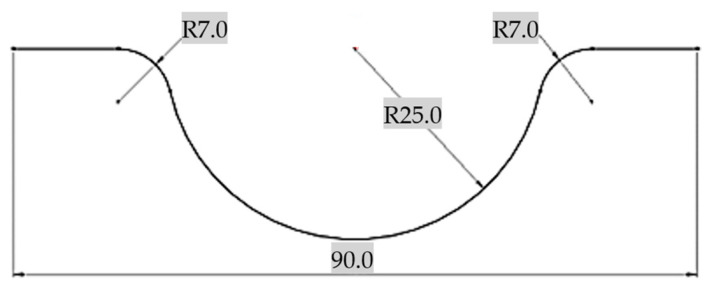
Ideal final part shape and dimensions used for modeling the initial punch 3D model.

An initial 3D model was designed using the final shape of the deformed part from Figure 2.

**Figure 3:**
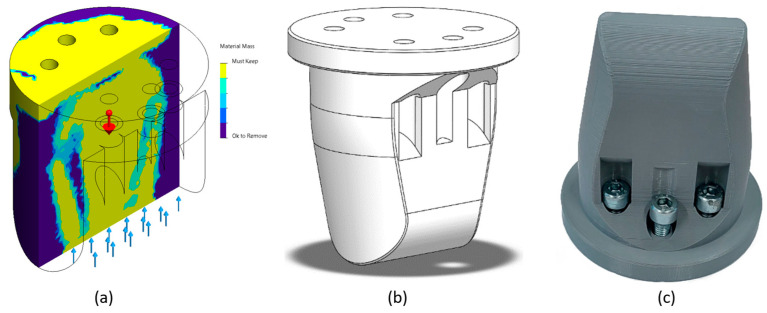
(**a**) Topology study on the initial 3D punch model performed in gravitational field (9.81 m/s^2^, indicated by the red arrow) and uniform distributed force on the contact surface (blue arrows); (**b**) resulting optimized 3D model, and (**c**) 3D-printed punch.

Using SolidWorks Simulation—Topology Study, the optimal shape for the punch construction for the forming process was determined, as indicated in Figure 3a. It explores the design iterations of a component that satisfy a given optimization objective and geometric constraints.

A topology study achieves the optimization of the nonparametric form of the parts. Starting from a maximum design space—which represents the maximum size allowed for a part—and considering all the applied loads, fasteners, and manufacturing constraints, the optimization of the topology seeks a new arrangement of the material within the limits of the maximum permitted geometry, by redistributing the material. The part thus optimized meets all the mechanical and manufacturing requirements (Figure 3b).

In addition to the optimization objective, design constraints, such as the maximum deformation, percentage of mass removed, and manufacturing processes, are defined to ensure that the required mechanical properties are met. For a successful topology study, the design proposal reached by the iterative optimization process meets all the structural and manufacturing requirements introduced.

For the configuration of the study, the following characteristics were imposed.

1. The primary purpose of optimization leads to the optimization algorithm’s mathematical formulation. In this case, the best rigidity-to-weight ratio was chosen, where the algorithm tries to minimize the overall conformity of the model, which is a measure of the rigidity’s overall (mutual) flexibility. The sum of the deformation energies of all elements defines the conformity.
2. Limiting constraints for solutions in the design space. In this case, the percentage of the mass to be eliminated was chosen (max. 30%).
3. Preserved areas excluded from the optimization process and retained in their final form. Geometric features in which loads and fasteners are applied are preserved by default. In this case, all the fixing holes of the punch on the dynamometer and the contact surface with the metal sheets were preserved.
4. The geometrical constraints imposed by manufacturing processes ensure that the optimized part is manufacturable. These constraints, such as the ejecting direction, thickness control, or symmetry control, are not required in this case, the part being constructed by additive manufacturing, as seen in Figure 3c.

Depending on the setting of the optimization objectivate, the manufacturing control, the network of elements, and the loading and limiting conditions, the optimization process results in a suitable design derivative of the initial maximum design space.

**Figure 4:**
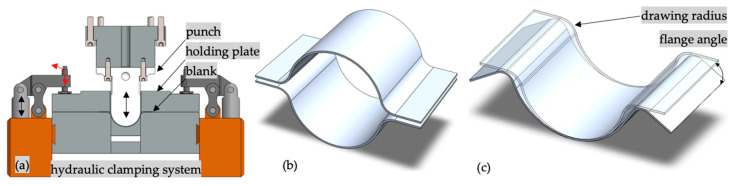
(**a**) Punch-forming setup cross-section, indicating the main components and their movements (black arrows indicate linear displacement of the hydraulic press main column and red arrows indicate angular displacement of the clamping system); (**b**) longeron assembly; (**c**) punch-formed longeron component and measured parameters.

A schematic representation of the punch-forming process and part geometry is presented in Figure 4a,b. The material blank is retained between the holding plate and the main body of the clamping system. Hydraulic pistons press on the holding plate, assuring the required tightening pressure. The framework for this experimental study is related to the structural behavior of tubular structure analyses at impact [26]. The authors’ previous experimental research indicated that assembling the longeron from Figure 4b by welding or adhesive is conditioned by the flange angle and drawing radius. It is evident that considering the elastic springback, the part’s final shape differs from the ideal shape by a certain amount; see Figure 4c. An improvement to the drawing process was needed, which led to the development of the adaptive punch-forming algorithm (A.P.F.A.). Considering the flange angle and drawing radius shape improvement, the material must flow with specific amounts at any step in the punch-forming process.

One aspect that emerges from this clamping system is the localization of high-stress areas between the drawing radius and flange angle; as the punch deforms the material, it can be stretched to the point at which shearing occurs in the direction of the formed bending radius. Furthermore, the flange deformation and elastic springback are excessive if no shearing occurs. One solution to this problem is allowing the material to flow by regulating the clamping pressure with which the blank is held. Analyzing the process leads to constructing a solution that allows automatic control of the hydraulic pressure of the clamping system by considering, at any point in the process, the punching force, speed, and stroke.

The punch-forming process, proposed by the authors in this experimental study, seeks to highlight the use of a 3D-printed punch along with a comparison between a standard pre-programmed and an adaptive one. It implies deforming 120 mm × 50 mm aluminum 6061-T4 blanks (AMAG, Ranshofen, Austria, with the chemical composition and mechanical properties given in Table 1) while measuring the punch force, speed, stroke, clamping system pressure, drawing radius, flange angle, and process time. 

**Figure 5:**
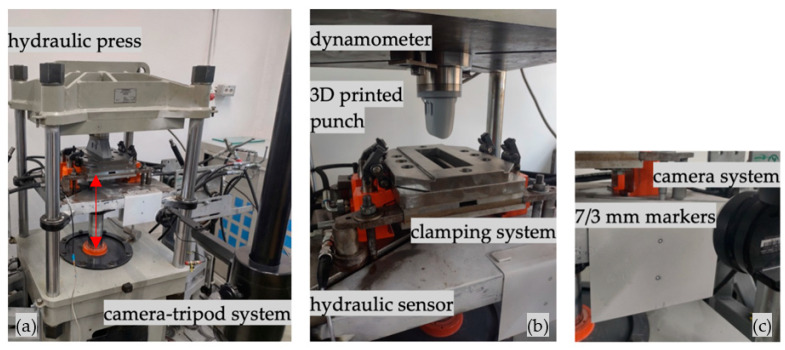
Experimental setup of the punch-forming process indicating (**a**) hydraulic press and camera tripod system (indicated with the red arrows the linear displacement of the hydraulic press main column); (**b**) dynamometer, 3D-printed punch, clamping system, and hydraulic sensor; and (**c**) 7/3 mm markers.

Figure 5a–c highlight the general methodology from an industrial equipment perspective. A Hydramold hydraulic press (Hydramold, Iași, Romania) was used as base equipment as its main control panel allows pre-programing and real-time control; a key advantage is that the stroke can be digitally controlled and measured, being displayed on a screen. On the upper plate of the hydraulic press, a Kistler 9272 dynamometer (Kistler Holding AG, Winterthur, Switzerland) is mounted, which also acts as a fastening system for the 3D-printed punch. The hydraulic clamping system is an integrated part of the hydraulic press; a downside of the mechanism is that the pressure is manually controlled. As the digital nature of the proposed solution implies real-time control of each parameter, the hydraulic pressure is assured separately by an Ecoroll HGP 3.0 hydraulic pump (Ecoroll AG, Celle, Germany); this configuration allows for digital control of the clamping system’s hydraulic pressure, through a solenoid valve and hydraulic sensor. 

Although the hydraulic press measures the stroke, this is an internal measurement and does not offer the possibility of serial communication. Therefore, a computer vision measuring system was developed based on the authors’ previous work [14]. As described in the general methodology [14], the system tracks calibrated white-on-black 7/3 mm markers fixed on a plate that moves at the same pace as the clamping system. The measurement technique was validated by comparing its results to the hydraulic press’ internal measurement system; the maximum measurement error was 0.714%, representing a deviation of 0.2 mm for a 28 mm stroke.

**Figure 6:**
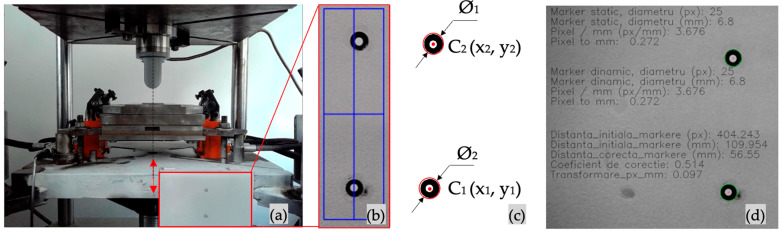
(**a**) Experimental setup side view indicating the hydraulic press movement and marker position (indicated with the red arrows the linear displacement of the hydraulic press main column); (**b**) region of interest (ROI) user selection; (**c**) image transformation result, indicating the diameter and center of circle coordinates; (**d**) software information of the auto-calibration coefficient, also used for user interpretation.

The script, used for computer vision capabilities, is written in Python (Python Software Foundation, Wilmington, Delaware, Statele Unite, 2022, version 3.7) and uses the OpenCV library [27]. The markers were placed on a metal plate, and the center-to-center distance was measured using a GOM Atos 3D measuring system. Thus, an auto-calibration function was implemented to measure the marker’s distance and diameter, resulting in a calibration coefficient. The program initiates with 1 bar pressure in the clamping system to ensure that the blank is held while the punch is placed 0.1 mm above the material. In Figure 6a, the camera view is displayed using the red rectangle; the next step in the process is for the user to select the region of interest (ROI), indicated in Figure 3b by a blue rectangle; until the end of the script, only this region will be analyzed. The frames contained in the ROI are transformed until the background is changed to white; at this stage, as in Figure 6c, the circle boundary is determined (Ø_1_ and Ø_2_), and therefore the origin (*C*_1_ and *C*_2_) and its coordinates (*x*_1_, *y*_1_, and *x*_2_, *y*_2_), in pixels and relative to the lower left part of the image. The distance between *C*_1_ and *C*_2_ is calculated with Equation (2), and the result is converted from pixels to mm using the calibration coefficient, as indicated in Figure 6d.

(2) $$\left(C_{1},C_{2}\right)=\sqrt{{\left(x_{2}-x_{1}\right)}^{2}+{\left(y_{2}-y_{1}\right)}^{2}}$$

**Figure 7:**
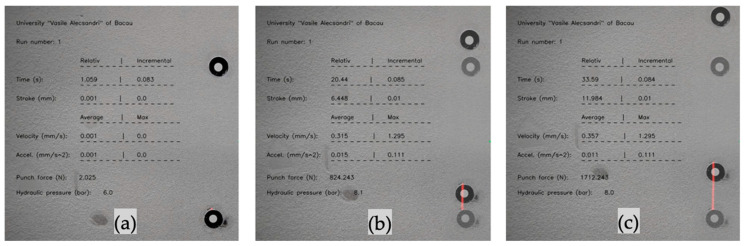
(**a**) User interface of the speed prediction algorithm initialization; (**b**) measured and calculated process parameters along with the (**c**) lower marker trajectory (indicated by the red line).

Once the center-to-center circle distance is determined, the algorithm initiates the control of the hydraulic press pump’s solenoid valve, thus controlling the punch stroke and hydraulic pressure; if the system is on self-control, an additional function connects to the Kistler’s data acquisition software DewesoftX (Dewesoft, Trbovlje, Slovenia, 2022, version 4) and retrieves real-time force measurements. From this point on, only the lower marker’s initial and actual position is used to keep track of its trajectory, as indicated in Figure 7a–c. In addition, the output image information, such as the run number, elapsed time, stroke, velocity, acceleration, punch force, and hydraulic clamping pressure, is displayed as it changes in real time; these values are then stored in .csv files.

Initial tests indicated that the forces needed to deform the aluminum blank reached 20 kN; as a result, the punch was subjected to high contact pressure. Therefore, ensuring the structural integrity through all the tests implied using a tough material such as the BASF Ultrafuse PLA PRO1 3D printing wire (BASF, Ludwigshafen am Rhein, Germany); the mechanical properties of the material are indicated in Table 2, and the printing parameters are indicated in Table 3.

### 2.2. Software Solution and Experimental Plan

**Figure 8:**
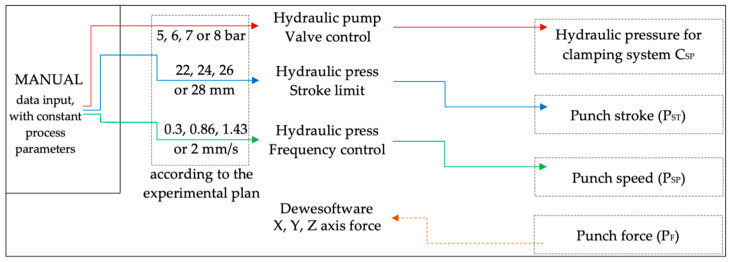
Manual data input script logic that uses pre-determined, constant process parameters, indicating with arrows the control flow.

A high degree of confidence in the accuracy of the software solution was required so that the hydraulic press and pump could be controlled with minimal or no human intervention. Therefore, the adaptive punch-forming algorithm (A.P.F.A.) had to be verified from the point of view previously described while offering improvements to the process. The following modes were implemented to compare and understand how to compile the algorithm: manual data input with constant process parameters and variable data input with variable process parameters. The first case implies a pre-programmed process with a specific experimental plan, as indicated in Figure 8; data are directed in one direction, a command is given, and a measurement is made.

**Figure 9:**
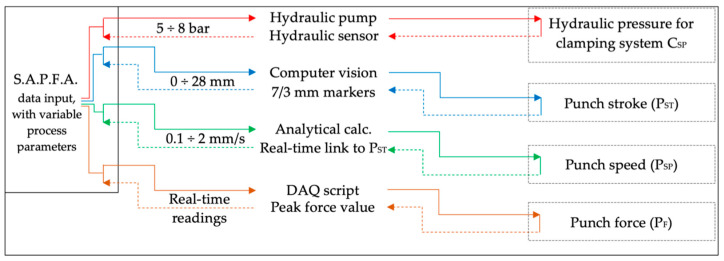
A.P.F.A. data input script logic that uses variable process parameters, indicating with arrows the control flow.

The proposed algorithm offers further functionality, as the communication is bidirectional (Figure 9); the measurements determine the commands to keep the punch force and hydraulic clamping pressure as constant as possible, considering the stroke increase. It can be noted from Figure 8 and Figure 9 that there are lower and upper limits for the hydraulic clamping system, stroke, and punch speed. Previous tests indicate in the case of the hydraulic pressure that pressure of less than 5 bar does not offer enough clamping force, while pressure above 8 bar results in shearing of the parts. A stroke of 22 mm is required for the part to be drawn in shape, while a value above 28 mm leads to excessive punch forces. A speed of 0.3 mm/s is the minimum speed of the hydraulic press, and 2 mm/s is the maximum that it can offer. Therefore, considering this limitation and the results obtained from the manual data input for the deformed parts, the A.P.F.A. was constructed to adjust each process parameter as the speed, pressure, stroke, and punch force are mathematically correlated. The correlation was implemented following the results obtained by analyzing the manual data input using the variance analysis (ANOVA) methodology. The plan proposed for this experimental study is, in consequence, tailored for each data input mode. In the case of the manual data input, 20 parts were analyzed, while, for the variable data input, we used 9.

### 2.3. Measurement Methodology

**Figure 10:**
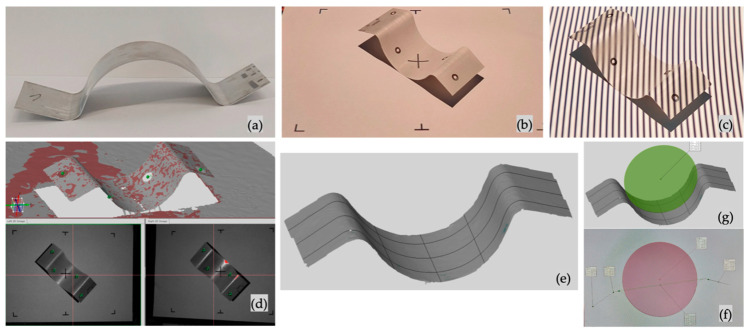
(**a**) Punched-formed part anti-reflex-coated; (**b**) part prepared for measuring, placed in the center of the scanning are, and (**c**) through the measuring process; (**d**) resulting mesh from the 3D measuring system; (**e**) intersection lines between parts with longitudinal and cross planes; (**f**,**g**) represent the measured areas.

The GOM Atos II 400 3D measuring system (Carl Zeiss GOM Metrology, Braunschweig, Germania) was used to measure the flange angle and drawing radius. Figure 10a–f highlight the measurement methodology used for this experimental study. The parts were sprayed with an anti-reflexive coating MR Chemie 2000 (MR Chemie GmbH, Unna, Germania) to avoid shiny spots on the parts and gaps in the measured area. The markers were cleaned, and the part was rotated between 5 and 8 angles for one complete measurement. With the help of the measuring system’s software, the scanned geometry was cleaned, purged, and transformed into a mesh. Taking as a reference the plane on which the parts were positioned, parallel planes were constructed transversally and longitudinally, keeping a constant distance between them. The intersection between these planes and the parts resulted in intersection lines, as indicated in Figure 10e. Each value is, therefore, an average of three measurements.

## 3. Results

From an industrial point of view, a part can enter production when the required dimensions, deviations, surface finish, and material hardness are within their tolerance ranges. When a part is at the first article inspection (F.A.I.) stage, changes must be made to the manufacturing process so that the part respects the design requirements. Therefore, constant changes to the process parameters are consistently correlated with part measurements. It is a common practice in any production process for these adjustments to be performed. As manufacturing time is a critical factor in any industrial process, any additional step is unwanted, but, if necessary, it must be as short as possible.

The Results section highlights the algorithm’s utility and is divided into pre-programmed and A.P.F.A. modes. The ANOVA methodology was conducted using the Design Expert v.12 software to determine if a mathematical connection between the process parameters and the result could be established. The results are relevant for determining a significant link between the input and output process parameters and understanding the direction that the A.P.F.A. algorithm should take. 

In terms of an ANOVA analysis, statistical analysis is performed. The interpretation of the outcome is related to terms such as the following:

- F-value (calculates the variance between means that differ significantly, indicating that the results did not occur by chance);
- *p*-value (correlated to the F-value; a value lower than 0.05 indicates the statistical significance of the observed results);
- R-squared (R^2^; values higher than 0.9 indicate high confidence in the model’s capacity to predict a valid response) and its variations:
  ○ Adjusted R^2^ (Adj-R^2^)—the amount of variation about the mean indicated by the model;
  ○ Predicted R^2^—measures how the model predicts a response.

In terms of experimental precision, the adjusted R^2^ and predicted R^2^ difference should not be greater than 0.2, as indicated by the official documentation [28]. 

Another critical aspect is the signal-to-noise ratio; it is evaluated by the adequate precision coefficient (Adeq. Precision), with a ratio greater than 4 being desirable.

### 3.1. Pre-Programed Controlled Process Results

The pre-programmed process follows a predictable path in terms of input process parameters. Indicated in Table 4 is the experimental plan along with the related measurements (maximum punching force, drawing radius, flange angle, and process time). The design factors are the clamping pressure (5, 6, 7, 8 bar), punch velocity (0.3, 0.866, 1.433, 2.0 mm/s), and stroke (22, 24, 26, 28 mm). 

**Figure 11:**
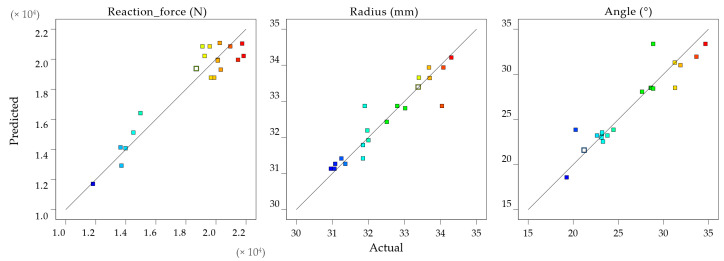
Predicted vs. actual data plots for reaction force, drawing radius, and flange angle, indicated as color range distribution (from blue squares for lower values to red squares for higher values).

Measurements were introduced into the Design Expert software (State-Ease, Minneapolis, MN, USA, 2022, version 12), and analyzed. The first step in the data validation process is the predicted vs. actual data plots from Figure 11, which visually interpret the predicted data in correlation with the measured ones. A 45° reference line indicates well-predicted values; the prediction is less accurate as the distance from the reference line increases. The data for the reaction force, drawing radius, and flange angle are near the reference line, indicating that the overall mathematical model can accurately make predictions.

The ANOVA analysis indicates that each model is significant. Table 5 shows the *p*-value for each factor model, which is less than 0.05, indicating that the data were not obtained by chance; thus, the mathematical connection is reliable. Furthermore, each input process parameter (pressure, velocity, and stroke) is significant for the outcome.

From a statistical point of view, the data indicate that the maximum punch force F-value of 15.95 implies that the model is significant. There is only a 0.01% chance that an F-value of this size could occur due to noise. Regarding the drawing radius, the model F-value of 6.58 implies that the model is significant. There is a 0.46% chance that an F-value of this size could occur due to noise. The flange angle indicates the same tendency, as an F-value of 15.60 indicates that the model is significant. The data suggest a 0.01% chance that it can occur due to noise.

Extensive statistical data analysis involves considering the input values’ interaction with the outcome. The maximum punch force *p*-value is 3.35 × 10^−5^, less than 0.05, indicating that the model terms are significant. A, B, C, BC, and AB^2^ are significant model terms in this case. The punch velocity model *p*-value is 0.004564, indicating that the model terms are significant. The factors and their interaction A, B, C, AC, A^2^, B^2^, C^2^, A^2^C, and AB² are significant model terms. For the flange angle, the calculated *p*-values of 2.93 × 10^−5^ also indicate significant model terms. B, C, AB, BC, and A^2^ are significant model terms in this case.

Another essential aspect of the analysis is the lack of fit F-value; a non-significant lack of fit is good, as it indicates that the model fits. In the case of the maximum punch force, punch velocity, and stroke, this value is 1.46, 0.23, and 0.36, implying that the lack of fit is insignificant relative to the pure error. For all the factors, there is a 35.08%, 8.93%, and 9.88% chance, respectively, that a lack of fit F-value of this magnitude could occur due to noise.

The Adj-R^2^ and predicted R^2^ need to be 0.2 units apart in correlation with the ANOVA statistical analysis indications. The adjusted R^2^ values are in reasonable agreement with the predicted R^2^, as indicated by Table 5 for the maximum punch force (0.7089 and 0.8464), punch velocity (0.5614 and 0.7461), and punch stroke (0.7214 and 0.8217). Each factor’s (maximum punch force, punch velocity, and stroke) signal-to-noise ratio is greater than 4, indicating an adequate signal. Thus, the model can be used to navigate the design space.

**Figure 12:**
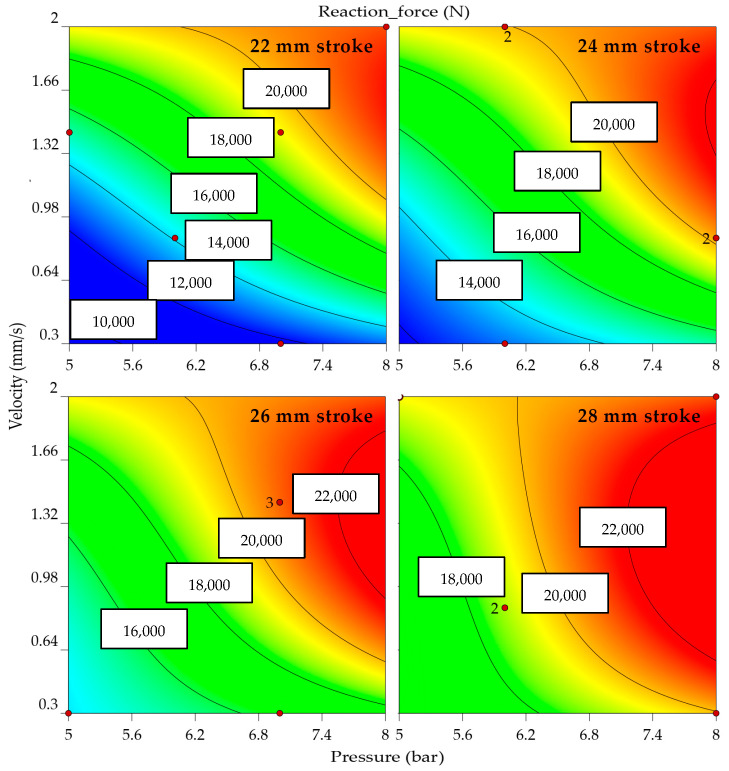
Reaction force by velocity, pressure, and stroke (indicating part of the input data with red dots and the distribution of values by color range, where blue is the minimum predicted value and red is the maximum; the numbering adjacent to the red dots indicates the number of center points replication).

**Figure 13:**
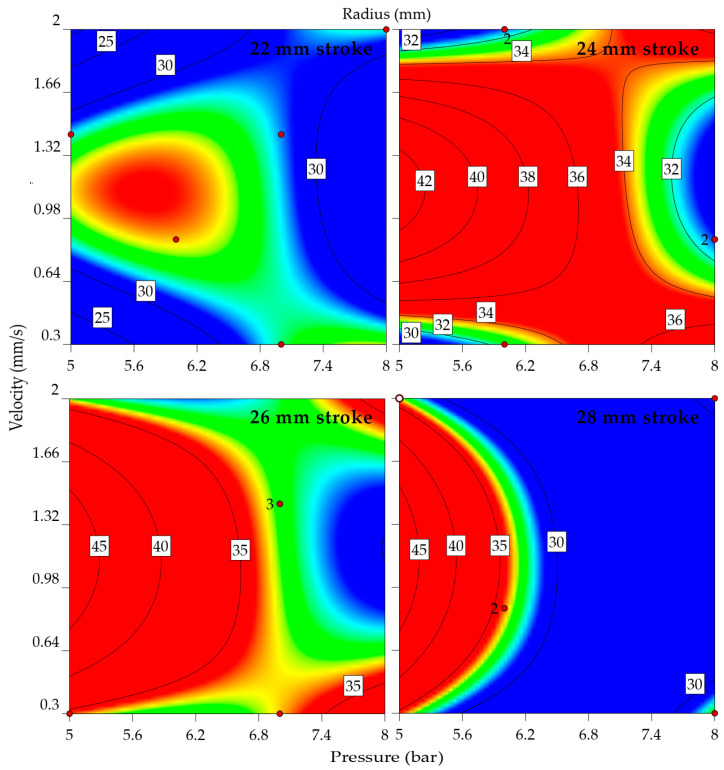
Drawing radius by velocity, pressure, and stroke (indicating part of the input data with red dots and the distribution of values by color range, where blue is the minimum predicted value and red is the maximum; the numbering adjacent to the red dots indicates the number of center points replication).

**Figure 14:**
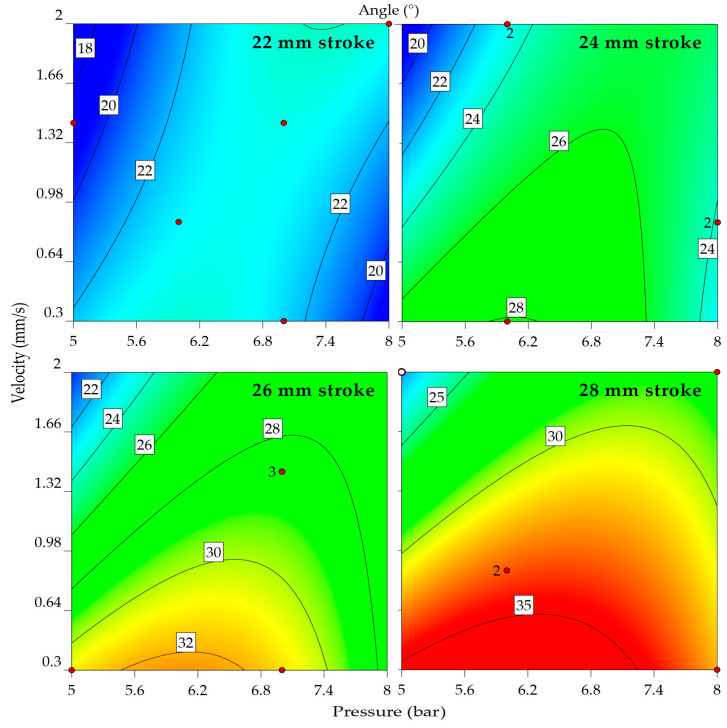
Flange angle by velocity, pressure, and stroke (indicating part of the input data with red dots and the distribution of values by color range, where blue is the minimum predicted value and red is the maximum; the numbering adjacent to the red dots indicates the number of center points replication).

These mathematical models’ predictions are accurate as the models are significant, as the R^2^ values are close to 0.9. The correlation functions are indicated in Equations (2)–(4) as second-degree polynemes for each factor: maximum punch force, drawing radius, and flange angle. The equations are a starting point in the design of the adaptive algorithm. Moreover, they are used to generate the model graphs for the reaction force (Figure 12), drawing radius (Figure 13), and flange angle (Figure 14).

(3) $$F=-21823.2-12.05P-4592.4V+1384.5S+4461.3PV-695.5VS+11950V^{2}-2089.2PV^{2}$$

(4) $$R=-1282.1+269.4P-155.9V+65.9S-22.3PV-10.7PS-18.5P^{2}-67.9V^{2}-0.5S^{2}+0.7P^{2}S+9.6PV^{2}$$

(5) $$A=-73.1+15.03P+4.5V+2.4S+2.01PV-0.8VS-1.3P^{2}$$

A color gradient representation of the interaction of the factors concerning each outcome visually highlights the result (blue being used for low-intensity outcomes while red is for the extreme ones). As expected, the punching force directly depends on the punching stroke, velocity, and clamping pressure. When analyzing the graphs from Figure 10, for the 24, 26, and 28 mm strokes, the bending radius tends to increase as the clamping pressure decreases. In the case of the 22 and 24 mm strokes, the extreme values of the punch velocity lead to similar results. A lower punch velocity, in combination with average clamping pressure, offers high flange angles. The ideal shape of the part would be with lower values of the drawing radius and flange angle. An overall view is formed from these experimental data, as the algorithm should control the punch force, and, considering each step of the stroke, it should decrease the punch velocity and clamping pressure.

### 3.2. Adaptive Punch-Forming Algorithm-Controlled Process Results

As indicated in Figure 6, the A.P.F.A. data input script logic uses variable process parameters. It tries to find the optimum solution (parameters) at each stroke step, considering the mathematical models implemented from the pre-programmed analysis. Table 6 shows that the average pressures and velocities that the algorithm uses vary around 7.44 bar and 0.238 mm/s, respectively. The stroke value tends to be higher than defined, with an average of 0.174 mm, but this is due to the system’s inertia; nevertheless, in each case, the average error is 1%. In the case of the drawing radius and flange radius’ average values obtained with the pre-programmed process, the data indicate a minor improvement of 1.8% for the radius and 27.05% for the angle; this comes with less punch force, a 36% drop, from an average of 18.27 kN to 11.66 kN, while there is an increase in the processing time from 35.6 s to 107.6 s.

**Figure 15:**
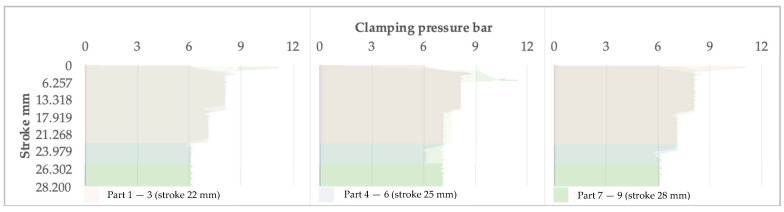
The evolution of the clamping pressure as the stroke increases is presented in groups depending on the indicated maximum stroke.

**Figure 16:**
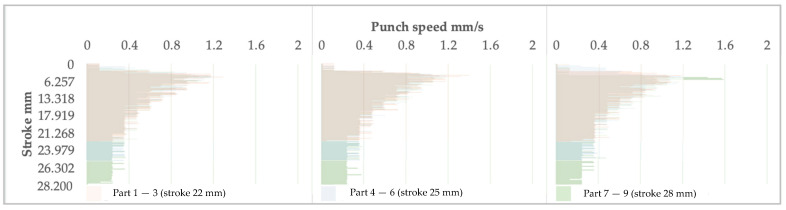
The evolution of the punch speed as the stroke increases is presented in groups depending on the indicated maximum stroke.

**Figure 17:**
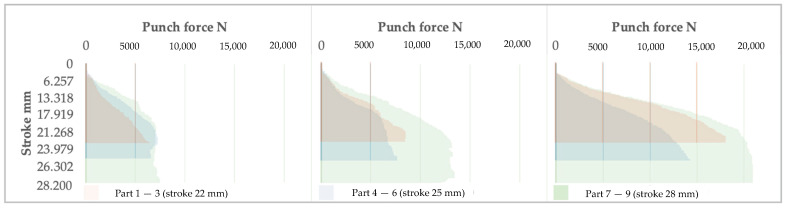
The evolution of the punch force as the stroke increases is presented in groups depending on the indicated maximum stroke.

Figure 15, Figure 16 and Figure 17 show the relationships between the stroke and the clamping pressure, the speed of the punch, and the punch force, respectively.

Regardless of the stroke, the graphs’ general shapes are similar, indicating that the system reacts concerning the three elements analyzed. 

In the case of the 22 and 28 mm strokes, the system adjusts the clamping pressure of the blank in stages. Firstly, the pressure increases to some maximum value; once the blank is stretched and forming is initiated, the pressure decreases to the level set by the system. Moreover, the strain rate decreases from the maximum value of 1.2 mm/s with the increased distance traveled by the punch. The trend is kept relatively constant until the end of the process.

Regarding the 24 mm stroke, the system adjusts the holding pressure in four stages; these are shorter in duration. The transition from one level to another is within roughly the same range as in previous cases. The situation is similar for the 26 mm stroke.

This phenomenon can be explained by the fact that the system needs more information accumulation at an overly low or high value of the stroke. Therefore, there is a delay in triggering the command to change the clamping force. It can be correlated with the data acquisition speed and the data processing speed of the system setup.

Graphically, the punch speed decreases parabolically since the clamping force must decrease following the desire to keep the strain ratio constant as a function of the forming stroke. It occurs similarly regardless of the forming mode.

With the increase in the forming depth, the inevitable increase in resistance to deformation occurs, caused by material hardening. This fact causes the forming force to increase, as shown in Figure 17.

## 4. Discussion

Given that the factors that decisively influence the plastic-forming processes of sheet metals are among the most varied, ranging from the chemical composition and structure of the material to the geometry of the parts, equipment, and the deformation conditions, this study proposes the use of an adaptive algorithm to control work equipment. Tools indispensable to the forming process have been obtained through additive manufacturing, thus providing additional freedom in correcting their active geometry.

The geometry of the tools is highly dependent on the springback of cold-formed sheet metal parts. The problem can be somewhat solved by addressing several work directions. This study focuses on a statistical analysis of a few influencing parameters that cause essential changes in part geometry after removing forming forces. However, this is different from the purpose of this study.

Thus, one of the solutions considered in this study is allowing the material to flow by regulating the clamping pressure with which the blank is held. Analyzing the process leads to the construction of a solution that will enable automatic control of the hydraulic pressure of the clamping system by considering, at any point in the process, the punching force, speed, and stroke.

By creating an algorithm that communicates bidirectionally, the measurements determine a command that keeps the forming and clamping forces constant as the stroke increases to the pre-set value.

The results were filtered through statistical analysis to highlight the mathematical link between the process parameters and the data obtained.

Considering the parameters of the forming process, its pre-programmed stage has a predictable trajectory. The data obtained and presented in graphical form show minimal differences between the estimated and measured values regarding reaction force, drawing radius, and flange angle. Thus, reasonably accurate estimates can be made through a general mathematical model. Moreover, ANOVA analysis suggests that each model is statistically significant, calculating a *p*-value of less than 0.05, demonstrating that these values were not obtained by chance. Further analyses yielded clear percentage values showing that noise did not influence the results. A significant factor in statistical data analysis is the lack of fit F-value. The model fits if there is a non-significant lack of fit.

The mathematical estimates of the models are accurate due to their statistical significance, the correlation functions being, in turn, the starting points in the design of the adaptive algorithm. Moreover, the graphs of the models—where blue is used to show weak correlations and red highlights strong correlations—are also obtained based on these equations.

In the case of the adaptive punch-forming algorithm-controlled process, the data input script logic uses variable process parameters, i.e., for each increment in the deformation stroke, the system tries to find the optimal process parameters by using previously implemented mathematical models.

**Figure 18:**
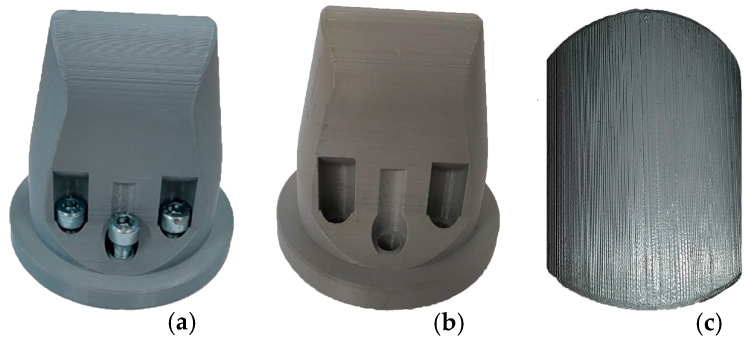
Comparison between the contact surface of the 3D-printed punch before (**a**) and after the (**b**) punch-forming process, indicating (**c**) the top view, after usage, highlighting the printing layers.

Another aspect of the process relates to the small degree of wear of the 3D-printed punch. It has to be considered that it was used to carry out initial tests, for the calibration of the machine and its correlation with the video detection system, and to obtain the samples for the experimental study. As can be noted in Figure 18a,b, no deformation has occurred, with the punch maintaining its initial shape. Furthermore, the top view of the 3D-printed punch, as can be noted from Figure 18c, indicates that it retained the roughness resulting from the printing process. This is an indicator that the stress induced by the contact pressure was distributed across the surface and was not concentrated. 

## 5. Conclusions

Following the analysis of the data collected from the experimental tests and the evaluation of the mathematical models used by the adaptive system, some conclusions can be drawn as follows:

- the reaction force of the system, depending on the forming speed, pressure, and stroke, increases rapidly after exceeding a depth of 25 mm;
- the radius of the part is strongly influenced by the clamping force, having inconsistent values at lower values;
- high values of the forming speed produce similar effects regarding the radius.

Thus, the three essential process parameters are changed accordingly depending on the drawing stroke. Although the general shape of the correlation graphs is similar, it can be concluded that the system reacts according to the mathematically estimated need in a precise way. Thus, the holding force is generally adjusted in three stages regardless of the prescribed deformation stroke. The forming speed decreases parabolically with the increase in the forming stroke, thus implying a proportional increase in the forming force.

The result obtained indicates that future research in this field can be directed toward training neural networks for decision making and investigating more alloys, complex shapes, and process parameters. Moreover, using machine learning and deep learning, an optimal punch shape can be obtained, for complex shapes, if we take into consideration factors such as the punch topology and geometry to decrease the springback effect, the optimization of the printing parameters, and the tool wear over time.

## Figures and Tables

**Table 1:** Chemical composition and mechanical properties of 6061-T4 aluminum alloy.

**Chemical Composition wt.%**									
Al	Si	Fe	Cu	Mn	Mg	Cr	Zn	Ti	Other
97.4	0.66	0.4	0.22	0.15	0.9	0.16	0.07	0.03	0.01
**Rp_0.2_ N/mm^2^**			**R_m_ N/mm^2^**				**A %**		
156			267				21		

**Table 2:** Mechanical properties of BASF Ultrafuse PLA PRO1 3D printing wire, obtained by tensile and Charpy impact tests.

Rm N/mm^2^	E_PLA_ N/mm^2^	A %	K kJ/m^2^ (Unnotched)
48	267	21.9	20.4

**Table 3:** The 3D printing parameters of the punch, BASF Ultrafuse PLA PRO1.

**Layer** **Height mm**	**Initial Layer** **Height mm**	**Line** **Width mm**	**Wall** **Thickness mm**	**Top** **Thickness mm**
0.15	0.2	0.375	1	1
**Bottom** **thickness mm**	**Horizontal** **expansion mm**	**Infill** **density %**	**Infill** **pattern**	**Infill** **overlap %**
1	−0.015	50	triangles	15
**Flow** **%**	**Build plate temperature** **°C**		**Print speed** **mm/s**	**Wall print speed** **mm/s**
102	60		75	37.5
**Printing time**			**Part weight g**	
8 h:59 m			164	

**Table 4:** Pre-programmed process experimental plan and measurements.

Part Number	Pressure Bar	Velocity mm/s	Stroke mm	Max. Punch Force N	Drawing Radius mm	Flange Angle °	Process Time s
1	8	0.866	24	21,839	30.96	24.46	27.7
2	6	0.866	28	19,878	34.08	34.66	32.3
3	8	0.866	24	19,245	31.06	20.265	27.7
4	5	0.3	26	13,646	34.3	31.885	86.6
5	5	2.0	28	18,689	33.38	21.2	14.0
6	6	0.866	22	13,984	33.4	23.13	25.4
7	8	0.3	28	20,116	33.02	33.655	93.3
8	7	1.433	22	20,316	31.85	23.19	15.3
9	6	2.0	24	20,117	31.08	22.635	12.0
10	5	1.433	22	14,501	31.85	19.265	15.3
11	8	2.0	28	21,741	31.25	28.86	14.0
12	6	2.0	24	21,466	31.36	23.785	12.0
13	7	1.433	26	19,584	34.04	31.29	18.1
14	6	0.866	28	19,663	33.68	28.86	32.3
15	6	0.3	24	13,708	32.51	27.625	80.0
16	7	0.3	26	14,972	33.7	31.26	86.6
17	8	2.0	22	20,250	32	23.195	11.0
18	7	1.433	26	19,096	32.8	28.59	18.1
19	7	0.3	22	11,811	31.97	23.295	73.3
20	7	1.433	26	20,951	31.9	28.645	18.1

**Table 5:** Partial ANOVA results in terms of *p*-value, R^2^, Adj-R_2_, predicted R^2^, and adequate precision.

Factor	Maximum Punch Force	Drawing Radius	Flange Angle
Model	*p*-value (significant if less than 0.05)		
	3.35 × 10^−5^ (significant)	0.004564 (significant)	2.93 × 10^−5^ (significant)
Pressure (A)	0.000547	0.002147	0.223187
Velocity (B)	8.81 × 10^−6^	0.00637	0.002118
Stroke (C)	0.000573	0.039428	3.81 × 10^−6^
	R-squared		
	0.902	0.879	0.878
	Adj-R^2^		
	0.846	0.746	0.821
	Predicted R^2^		
	0.708	0.561	0.721
		Adeq. precision	
	11.869	7.341	12.852

**Table 6:** A.P.F.A. average process parameters—experimental plan and measurements.

Part Number	Avg. Pressure Bar	Avg. Velocity mm/s	Stroke mm	Max. Punch Force N	Drawing Radius mm	Flange Angle °	Process Time s
1	7.45	0.261	22.199	6431	31.97	18.66	85.0
2	7.46	0.263	22.194	8591	32.29	27.93	84.4
3	7.67	0.275	22.247	18,109	32.43	23.37	81.0
4	7.12	0.257	25.168	7250	32.81	18.77	97.8
5	7.20	0.241	25.186	14,196	32.20	19.05	104.6
6	7.14	0.221	25.193	8265	31.57	22.52	114.1
7	7.05	0.204	28.130	13,483	31.60	19.81	138.2
8	8.85	0.210	28.200	21,184	31.14	24.43	134.6
9	7.08	0.217	28.054	7471	31.40	13.09	129.0

## Data Availability

Not applicable.

## References

[1] Hetz P., Suttner S., Merklein M. (2020). Investigation of the Springback Behaviour of High-strength Aluminium Alloys Based on Cross Profile Deep Drawing Tests. Procedia Manuf..

[2] Kim H.-K., Kim W.-J. (2018). A Springback Prediction Model for Warm Forming of Aluminum Alloy Sheets Using Tangential Stresses on a Cross-Section of Sheet. Metals.

[3] Sun L., Cai Z., He D., Li L. (2019). Aluminum Alloy Sheet-Forming Limit Curve Prediction Based on Original Measured Stress–Strain Data and Its Application in Stretch-Forming Process. Metals.

[4] Teng F., Liang J., Wang S., Han Q. (2022). Effect of Axial Normal Stress and Bending Moment between Contact and Non-Contact Zone on Forming Accuracy for Flexible Stretch Bending Formation. Metals.

[5] Patil S.P., Fenard Y., Bailkeri S., Heufer K.A., Markert B. (2019). Investigation of Sheet Metal Forming Using a Rapid Compression Machine. Materials.

[6] Cinar Z., Asmael M., Zeeshan Q., Safaei B. (2021). Effect of Springback on A6061 Sheet Metal Bending: A Review. J. Kejuruter..

[7] Lawanwomg K., Hamasaki H., Hino R., Yoshida F. (2014). A Novel Technology to Eliminate U-bending Springback of High Strength Steel Sheet by Using Additional Bending with Counter Punch. Procedia Eng..

[8] Choudhury I.A., Ghomi V. (2014). Springback reduction of aluminum sheet in V-bending dies. Proc. Inst. Mech. Eng. Part B J. Eng. Manuf..

[9] Milošević N.Z., Sedmak A.S., Bakić G.M., Lazić V., Milošević M., Mladenović G., Maslarević A. (2021). Determination of the Actual Stress-Strain Diagram for Undermatching Welded Joint Using DIC and FEM. Materials.

[10] Aakash B.S., Connors J., Shields M.D. (2019). Stress-strain data for aluminum 6061-T651 from 9 lots at 6 temperatures under uniaxial and plane strain tension. Data Brief.

[11] Mehdi D. (2015). Effect of Silicon content on the Mechanical Properties of Aluminum Alloy. Int. Res. J. Eng. Technol..

[12] Borodulina S., Kulachenko A., Nygårds M., Galland S. (2012). Stress-strain curve of paper revisited. Nord. Pulp Pap. Res. J..

[13] Chen Y., Clausen A., Hopperstad O., Langseth M. (2009). Stress–strain behaviour of aluminium alloys at a wide range of strain rates. Int. J. Solids Struct..

[14] Grigoras C.C., Zichil V., Chirita B., Ciubotariu V.A. (2021). Adaptive Stretch-Forming Process: A Computer Vision and Statistical Analysis Approach. Machines.

[15] Cuan-Urquizo E., Barocio E., Tejada-Ortigoza V., Pipes R.B., Rodriguez C.A., Roman-Flores A. (2019). Characterization of the Mechanical Properties of FFF Structures and Materials: A Review on the Experimental, Computational and Theoretical Approaches. Materials.

[16] Spathopoulos S.C., Stavroulakis G.E. (2020). Springback Prediction in Sheet Metal Forming, Based on Finite Element Analysis and Artificial Neural Network Approach. Appl. Mech..

[17] Lal R.K., Choubey V.K., Dwivedi J., Kumar S. (2018). Study of factors affecting Springback in Sheet Metal Forming and Deep Drawing Process. Mater. Today Proc..

[18] Chongthairungruang B., Uthaisangsuk V., Suranuntchai S., Jirathearanat S. (2013). Springback prediction in sheet metal forming of high strength steels. Mater. Des..

[19] Grigoras C., Chirita B., Brabie G., Ciofu C. (2019). Experimental analysis of AZ31B magnesium alloy sheet failure using punch stretching. IOP Conf. Ser. Mater. Sci. Eng..

[20] Grigoraș C., Chiri B., Brabie G. (2019). Additive manufacturing of a stretch forming die using 3D printing technology. IOP Conf. Ser. Mater. Sci. Eng..

[21] Brancewicz-Steinmetz E., Sawicki J. (2022). Bonding and Strengthening the PLA Biopolymer in Multi-Material Additive Manufacturing. Materials.

[22] Walia K., Khan A., Breedon P. (2021). Polymer-Based Additive Manufacturing: Process Optimisation for Low-Cost Industrial Robotics Manufacture. Polymers.

[23] Fico D., Rizzo D., Casciaro R., Corcione C.E. (2022). A Review of Polymer-Based Materials for Fused Filament Fabrication (FFF): Focus on Sustainability and Recycled Materials. Polymers.

[24] Guessasma S., Belhabib S., Bassir D., Nouri H., Gomes S. (2020). On the Mechanical Behaviour of Biosourced Cellular Polymer Manufactured Using Fused Deposition Modelling. Polymers.

[25] Introduction to Additive Manufacturing: Part Three. https://www.totalmateria.com/page.aspx?ID=CheckArticle&site=ktn&NM=439.

[26] Ciubotariu V.A., Radu M.C., Herghelegiu E., Zichil V., Grigoras C.C., Nechita E. (2022). Structural and Behaviour Optimization of Tubular Structures Made of Tailor Welded Blanks by Applying Taguchi and Genetic Algorithms Methods. Appl. Sci..

[27] Bradski G. (2000). The opencv library. Dr. Dobbs J. Softw. Tools.

[28] Interpretation of R-Squared, Experimenters Frequently Ask the Question “What is a Good R-Squared Value? How Low Can It Be before the Results Are Not Valid?”. https://www.statease.com/docs/v22.0/contents/analysis/interpretation-of-r-squared/.

